# Personalised acromegaly treatment: implementing precision medicine in the Gulf States

**DOI:** 10.3389/fendo.2026.1951420

**Published:** 2026-10-13

**Authors:** Tarik Elhadd, Khaled Aldahmani, Mussa Almalki, Aisha Ekhzaimy, Raya Almazrouei, Zainab Dabbous, Zaina Rohani, Ali Alzahrani

**Affiliations:** 1 Qatar Metabolic Institute, Department of Medicine, Hamad Medical Corporation, Doha, Qatar; 2 Division of Endocrinology, Tawam Hospital & Sheikh Tahnoon Bin Mohammed Medical City (STMC), Al Ain, United Arab Emirates; 3 Department of Internal Medicine, College of Medicine and Health Sciences, United Arab Emirates University, Al Ain, United Arab Emirates; 4 Obesity, Endocrine, and Metabolism Center, King Fahad Medical City, Second Health Cluster, Riyadh, Saudi Arabia; 5 College of Medicine, Alfaisal University, Riyadh, Saudi Arabia; 6 Endocrinology & Diabetes Unit, Department of Medicine, College of Medicine and King Saud University Medical City, King Saud University, Riyadh, Saudi Arabia; 7 Department of Medicine, King Faisal Specialist Hospital and Research Center, Jeddah, Saudi Arabia; 8 Molecular Endocrine Section, Research and Innovation, King Faisal Specialist Hospital and Research Center, Riyadh, Saudi Arabia; 9 Department of Medicine, King Faisal Specialist Hospital and Research Center, Riyadh, Saudi Arabia

**Keywords:** acromegaly, biomarkers, Gulf States, pasireotide, personalised therapy, precision medicine, somatostatin receptors, T2-weighted MRI

## Abstract

Acromegaly management in the Gulf region is complicated by a high burden of metabolic comorbidity, frequent presentation with macroadenomas and suboptimal biochemical control in available regional cohorts. Conventional first-generation somatostatin receptor ligands achieve biochemical control in only 30-40% of patients, rates lower than those observed in European cohorts, necessitating precision medicine strategies. MRI signal and tumour granulation patterns represent the most widely available biomarkers in Gulf healthcare systems and enable precise treatment selection. These biomarkers may help predict poor first-generation therapy response while identifying patients who may benefit from pasireotide, an efficacious second-generation somatostatin receptor ligand with multi-receptor binding capacity. Implementation of biomarker-guided therapy in Gulf healthcare systems can optimize therapeutic outcomes while addressing regional challenges of delayed diagnosis and advanced disease presentation. Regional challenges specific to Gulf healthcare systems are identified and a practical biomarker-guided algorithm tailored for the GCC region is proposed to optimise treatment selection using readily available diagnostic tools. This approach is more appropriate for Gulf populations who present with more advanced disease characteristics that may benefit from precision-guided first-line treatment selection, representing a paradigm shift away from current guideline recommendations toward truly personalised medicine. This evolution from empirical to precision medicine represents a potential advancement for improving acromegaly management where complex metabolic phenotypes demand sophisticated treatment strategies.

## Highlights

- Regional challenge: Gulf states face unique acromegaly management challenges with increased metabolic comorbidities, suboptimal control rates (32.8-43.7%) and higher macroadenoma rates (82.2%) compared to global averages.
- Precision medicine tools: T2-weighted MRI signal and granulation pattern can help estimate fg-SRL responsiveness, but they should complement rather than replace clinical judgement.
- Treatment optimisation: Biomarker-guided treatment selection can potentially eliminate months of ineffective therapy by identifying patients unlikely to respond to first-generation somatostatin receptor ligands and enabling appropriate treatment as early as possible.

## Regional context and therapeutic challenges

Acromegaly presents unique challenges in the Gulf region, where clinical characteristics reflect both the global burden of this rare endocrine disorder and region-specific factors that complicate diagnosis and management. Recent data from Qatar demonstrates a prevalence of 1.90 per 100,000 population, consistent with global estimates, yet reveals concerning patterns of delayed diagnosis and advanced disease presentation (1). Studies from the United Arab Emirates show a high macroadenoma rate of 82.2%, significantly higher than the 75% global average (2). This elevated rate may result from diagnostic delays (3, 4), as larger tumours may indicate longer disease duration before recognition (5). This possible delay in recognition is compounded by limited disease awareness, with endocrinologists successfully identifying acromegaly in 70.6% of cases with available data, while other specialities were only marginally effective in detecting acromegaly (3.9%-7.8%) (2).

The metabolic burden of acromegaly is particularly pronounced in Gulf populations, with diabetes mellitus affecting 45-53% of acromegaly patients compared to 28.8% in European cohorts, while obesity prevalence reaches 84.5% (1, 2). Despite high surgical intervention rates (76.7%), biochemical control rates range only from 32.8-43.7%, falling short of the 63% global average, with over half of patients having persistent disease despite treatment (1, 2). Despite these suboptimal control rates, in clinical practice patients are often maintained on ineffective first-generation therapies for several months to years without switching to more appropriate alternatives. Current clinical practice is usually to switch patients uncontrolled on first-generation SSAs after a maximum of 3–6 months after dose and compliance optimisation (6), yet implementation of this recommendation remains suboptimal in real-life conditions (7–9).

## Current treatment limitations

The current approach to acromegaly management follows a “trial and error” methodology, with first-generation somatostatin receptor ligands (fg-SRLs; octreotide and lanreotide) used as first-line medical therapy regardless of patient and tumour characteristics (10, 11). This approach achieves biochemical control in only 30-40% of patients in prospective trials, leaving a substantial proportion with uncontrolled disease (10). The delay in controlling disease in non-responders can be quantified in years, as every modification in medical therapy requires several months for full evaluation (12). Delayed achievement of biochemical control may lead to irreversible complications, as some metabolic and cardiovascular comorbidities associated with prolonged GH excess may not fully resolve even after disease control is achieved (10, 13, 14).

Pegvisomant, a growth hormone receptor antagonist, offers an alternative approach. It rapidly decreases IGF-1 levels, achieving normalization in over 62% of patients after long-term treatment (10 years) (15), but does not act on the tumour itself and requires IGF-1 monitoring as the primary efficacy marker (10, 12). Albeit with only one year of follow-up, data suggests that on pegvisomant, headache, fatigue and joint pain appear to remain largely unchanged over time (16).

## Pasireotide: an opportunity in precision medicine

Pasireotide is a second-generation somatostatin receptor ligand that binds with high affinity to SST5 and has activity at SST1, SST2, and SST3 receptors (10). Unlike fg-SRLs, which primarily target SST2 receptors, pasireotide’s multi-receptor binding capacity allows it to be effective in tumours with different receptor expression patterns (12). Head-to-head studies have demonstrated pasireotide’s superior efficacy compared to fg-SRLs across all patient populations, regardless of tumour characteristics (17). Treatment guidelines generally recommend pasireotide in second-line therapy following failure of fg-SRLs (9). Approval varies by region. In the USA, pasireotide is approved for patients for whom surgery is inadequate or inappropriate. In the Gulf states it is approved as treatment for acromegaly whenever medical therapy is appropriate; and in other countries, including the European Union, it is reserved after failed surgery and when patients are inadequately controlled with a fg-SRL.

## Biomarkers for precision medicine implementation

### T_2_-weighted MRI signal

T_2_-weighted magnetic resonance imaging (MRI) signal has emerged as a crucial predictor with opposite implications for fg-SRLs and pasireotide (10, 12). Hypointense adenomas show better biochemical response to fg-SRLs, with 90% achieving >50% IGF-I reduction compared to 36% with non-hypointense tumours (18). Thus, hypointense adenomas are good indicators for response to first-generation SRLs, whereas pasireotide demonstrates efficacy in both hypointense and hyperintense tumours. The Ruiz et al. study found that MRI signal intensity was not associated with hormonal control with pasireotide, with approximately 60% of patients achieving complete IGF-I normalization regardless of T_2_ signal characteristics, making pasireotide an effective option for patients who have failed first-generation SRLs irrespective of tumour signal intensity (19). Machine learning-based texture analysis of T_2_-weighted images also demonstrates high accuracy, with 85% of macroadenomas classified correctly regarding SRL response, offering an important step toward implementing artificial intelligence in precision medicine (20).

### Receptor expression patterns

Higher SST2A expression is associated with a better biochemical response to the fg-SRLs, octreotide and lanreotide, which have very similar receptor-binding profiles (21). Lower SST2 messenger RNA expression was associated with relatively greater pasireotide effect in an *in vitro* head-to-head study, but this should not be presented as a validated clinical treatment rule (22). The SST2:SST5 ratio further differentiates treatment response, with a validated cut-off ratio of 1.3 demonstrating 88% sensitivity and 92% specificity in predicting disease control (20). A high SST2:SST5 ratio (>1.3) indicates better response to first-generation therapies, while a low ratio suggests potential benefit from pasireotide therapy (23, 24).

### Histopathological patterns: a key accessible biomarker in Gulf healthcare systems

Tumour granulation pattern is one of the most practical pathological biomarkers for predicting treatment response. Routine electron microscopy is not required: CAM 5.2 immunohistochemistry for low-molecular-weight cytokeratins can distinguish densely granulated tumours, which show diffuse or perinuclear staining, from sparsely granulated tumours, which show dot-like paranuclear fibrous bodies (20, 25). Densely granulated tumours generally show greater IGF-I reduction with a first-generation SRL, whereas sparsely granulated tumours show a more modest response (25). In contrast, sparsely granulated tumours may respond well to pasireotide, with a reported mean IGF-I reduction of 58% compared with 15.6% in densely granulated tumours (26). Thus, CAM 5.2 provides a practical alternative to ultrastructural classification where electron microscopy is unavailable.

Additional exploratory markers include Aryl hydrocarbon receptor Interacting Protein (AIP) expression and mutations, which serve as specific biomarkers for first-generation SRL response (26, 27). Low AIP expression significantly increases treatment resistance to first-generation SRLs (100% resistance vs 60% in high AIP expression), while AIP levels do not influence pasireotide response (26). In cases of AIP mutations, pasireotide has been found to be beneficial in AIP mutation positive patients resistant to fg-SRLs (27). E-cadherin expression demonstrates strong predictive value for first-generation SRL response, with high expression predicting better outcomes (65.4% sensitivity and 88.5% specificity at optimal cut-off levels) (24).

### Patient demographics

Personalised treatment begins with a complete clinical history and integration of clinical, biochemical and radiological features. Younger age, male sex, higher baseline GH and IGF-I concentrations, larger tumour size and T2 iso- or hyperintensity are associated with a lower probability of biochemical control with fg-SRLs (18, 28). These predictors should be considered together rather than used in isolation. The ACROFAST study, a multicentre trial comparing personalised versus standard treatment strategies, supports the feasibility of precision medicine in acromegaly (29).

## Precision medicine algorithms

### Current evidence-based approaches

The Kasuki-Gadelha algorithm uses patient and tumour characteristics to guide first-line treatment selection (10). Patients with clear features of fg-SRL resistance, including low SST2 expression, low AIP expression, a sparsely granulated pattern and T_2_ hyperintensity on MRI, have less than a 50% chance of achieving disease control with fg-SRLs (10). In such patients, a trial of fg-SRL therapy may only delay effective treatment, particularly as pasireotide has shown superior efficacy to fg-SRLs (6).

Alternative algorithms evaluate densely granulated, SST2 positive, low Ki-67 and MRI T_2_ hypointensity as predictive of fg-SRL response; with sparsely granulated, SST2 negative, MRI T_2_ hyperintensity, young patients and AIP mutations as predictive of fg-SRL resistance (20). Sparsely granulated patterns with low E-cadherin and low SSTR2, detectable SST5, low or absent AIP, and high Ki-67 would suggest pasireotide as preferred initial therapy (12).

In a recent review, Giustina and colleagues proposed an updated algorithm adapted for the management of “difficult” acromegaly patients (**Figure 1**). They recommended that for patients who did not respond to first-generation SRLs, if they were uncontrolled for a maximum period of 3 to 6 months after dose and compliance optimisation, they should be switched to pasireotide or pegvisomant (6). However, in practice, the time to effective treatments for patients who do not respond to SRLs are often quantified in years, as every modification in medical therapy requires months to be fully evaluated (12). Survivin has been identified as a potential marker of fg-SRL resistance, as low tumour expression is associated with poor treatment response (30). However, survivin immunohistochemistry is not currently applied in routine clinical practice and awaits validation (31).

**Figure 1:**
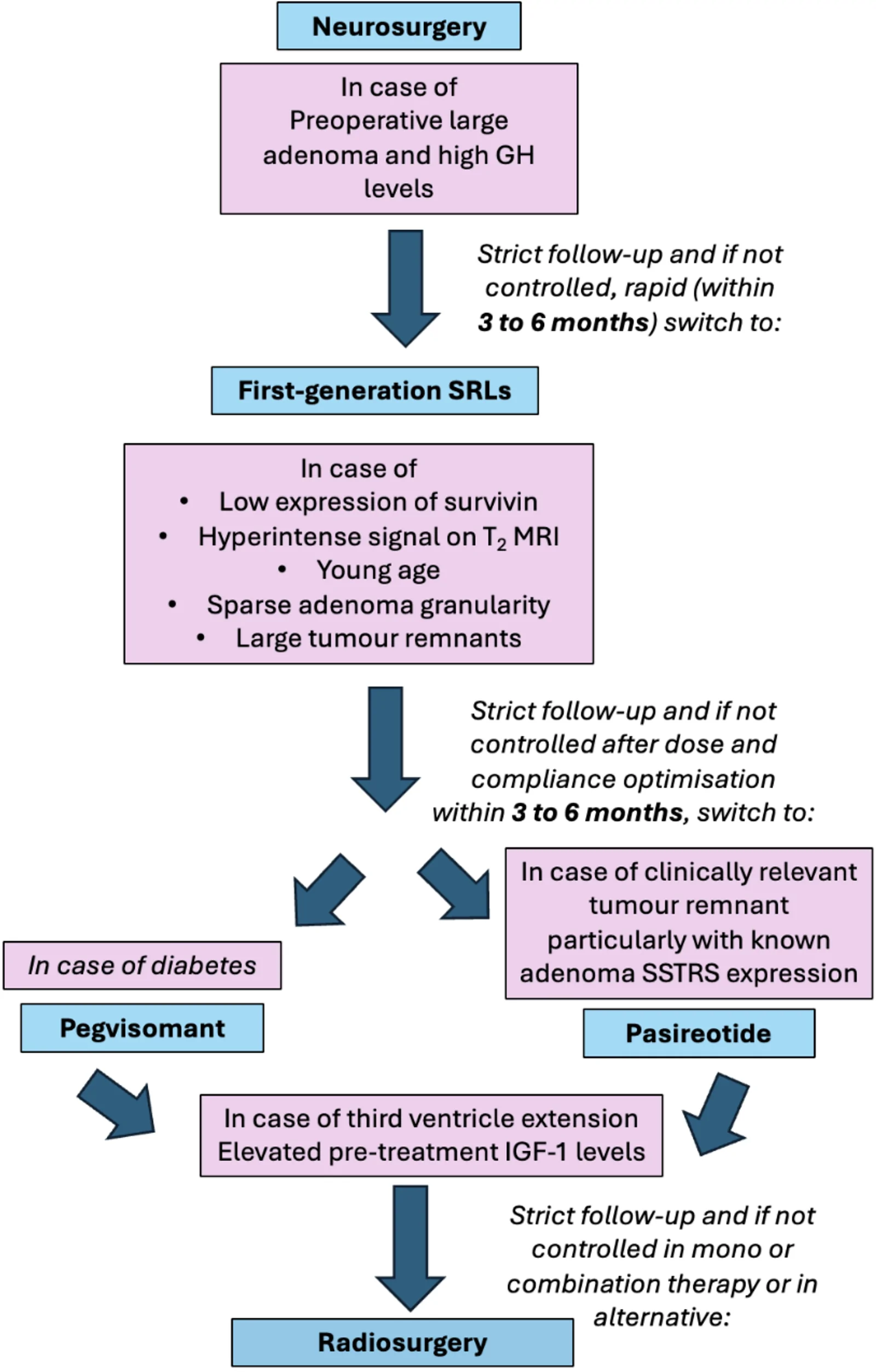
Approach to personalisation of acromegaly management integrating novel molecular and clinical biomarkers into existing guidelines. Adapted from Giustina 2023 (6).

Selection between pasireotide or pegvisomant should integrate tumour behaviour, biochemical severity and metabolic risk. A large or growing tumour may favour pasireotide when glycaemic risk is acceptable, whereas significant diabetes or high hyperglycaemia risk may favour pegvisomant when tumour growth is not a major concern (10). Combination therapy can be considered in selected resistant cases, but the evidence base is smaller and treatment burden and cost must be considered (12).

Given the particular challenges in the GCC region, including healthcare access disparities, complex referral systems, and varying levels of endocrine expertise across centres, these treatment delays are likely to be significantly longer than in resource-rich healthcare systems, making precision medicine approaches even more critical for optimal patient outcomes (2). This non-individualized approach thus not only prolongs the path to control but is inherently inefficient (29, 32).

The 2025 Acromegaly Consensus Conference reinforces the importance of individualised management based on clinical, imaging and pathological features (33). The consensus endorsed multiple predictive biomarkers, noting that adenoma granulation pattern assessment is considered a minimum requirement for determining SRL responsiveness, while SSTR2 immunostaining might predict response when available.^31^ This supports the practical biomarker-guided approach proposed for Gulf healthcare systems, where T_2_-weighted MRI signal characteristics and granulation patterns are the most widely accessible diagnostic tools for treatment selection. The consensus also emphasised that pasireotide LAR might be particularly beneficial in patients with relevant or growing adenomas who have a low risk of hyperglycaemia, aligning with the precision medicine strategy for a GCC-adapted algorithm (33).

### Artificial intelligence integration

Machine learning models incorporating SSTR2, SSTR5, granulation pattern, sex, age, and pre-treatment GH/IGF-1 have reported 86.3% accuracy, 83.3% positive predictive value and 87.5% negative predictive value (34). These machine learning models represent a significant advance in precision medicine, as they can simultaneously process multiple biomarkers to generate treatment recommendations with higher accuracy than clinical judgment based on individual parameters alone. Future development of mobile applications incorporating these algorithms could facilitate point-of-care treatment decisions in Gulf healthcare systems (10).

### Summary of treatment-specific patient profiles

First-generation therapies work best in patients with high SST2 expression, high SST2:SST5 ratios, dense tumour granulation, hypointense T_2_ MRI, high AIP expression, and older female demographics (10, 22, 28, 35). Conversely, low SST2 expression, low SST2:SST5 ratios, sparse tumour granulation and iso-hyperintense T_2_ MRI are suggestive of a potential resistance to fg-SRLs. In these resistant cases, pasireotide represents a more appropriate first-line treatment choice rather than pursuing months of ineffective first-generation therapy (10, 12).

## Gulf States implementation strategy

### Infrastructure requirements

Despite the potential of precision medicine treatment biomarkers, limited comprehensive studies and routine clinical practices make it challenging for clinicians to use these markers for patient selection, leading to a reliance on imaging and patient history (36). Although comprehensive data on biomarker availability across Gulf healthcare systems is limited, practical implementation should likely focus on the most widely accessible diagnostic tools including T_2_-weighted MRI and histopathological granulation pattern analysis, which are standard components of acromegaly workup in most tertiary centres. Advanced molecular testing (SST receptor expression, AIP mutations) may be restricted to specialized reference centres (35), similar to limitations observed in many European healthcare systems. However, emerging evidence suggests that even a limited combination of complementary biomarkers, particularly MRI signal characteristics (35), combined with granulation patterns, may provide clinically meaningful predictive accuracy for treatment selection, though this approach requires prospective validation (37, 38). Effective implementation requires standardised biomarker assessment methodologies across institutions, regional capabilities for advanced immunohistochemical analysis, healthcare provider training in precision medicine approaches, and establishment of reference centres for standardised analysis (11). Multicentre studies within the Gulf region should validate predictive algorithms in local populations and determine the optimal combination of accessible biomarkers for clinical decision-making.

### Regional considerations

Implementation requires addressing specific Gulf region challenges including high diabetes prevalence, advanced disease presentation, and healthcare access disparities (2, 8). While pasireotide may affect glucose metabolism (39), the benefits of achieving acromegaly control typically outweigh the risks of hyperglycaemia, which can be effectively managed with oral hypoglycaemic agents and dietary modifications in most patients (40). Controlled diabetes should not be considered a contraindication for pasireotide use when clinically indicated (36). Baseline characteristics that may influence glucose response include older age, dyslipidaemia, elevated BMI, and hypertension, requiring appropriate monitoring during treatment (41).

### Cost-effectiveness

Precision medicine approaches can reduce healthcare costs by eliminating ineffective treatments and accelerating time to disease control, particularly important given healthcare access disparities where high-tier insurance coverage predicts better outcomes (2). Given the efficacy of pasireotide, its use in acromegaly is expected to reduce healthcare costs by reducing comorbidities. This is likely to be even more pronounced in the era of precision medicine (34, 42). In real-world conditions, a recent study in Spain showed that pasireotide was significantly more cost-effective than pegvisomant in patients with first-line treatment failure (43).

### Clinical implementation guidelines

Current consensus guidelines recommend using T_2_ MRI intensity, somatostatin receptor immunopositivity, tumour granulation patterns, and AIP mutations for second-line treatment selection between pasireotide and pegvisomant (44). Evidence quality varies from moderate to very low for different predictors, emphasizing the need for continued validation studies (44).

Utilising available clinical and molecular parameters to guide treatment decisions facilitates personalised care delivery and optimises outcomes (10). The combination of validated biomarkers with regional healthcare considerations provides a framework for implementing precision medicine in Gulf healthcare systems.

### GCC-adapted implementation algorithm

Given regional differences in access to advanced pathology, a simplified precision-medicine algorithm should prioritise readily available clinical and radiological features (**Figure 2**). Age, sex, baseline GH and IGF-I concentrations, tumour size and invasiveness, and T2-weighted MRI signal should be assessed before treatment selection. When surgical tissue is available, CAM 5.2 immunohistochemistry can classify the granulation pattern without routine electron microscopy. A younger patient with high biochemical activity, a large T2 iso- or hyperintense tumour and a sparsely granulated pattern is less likely to respond to fg-SRLs and may merit earlier consideration of an alternative treatment, while recognising that these are probabilistic predictors and do not replace clinical judgement (10, 18, 25). 

**Figure 2:**
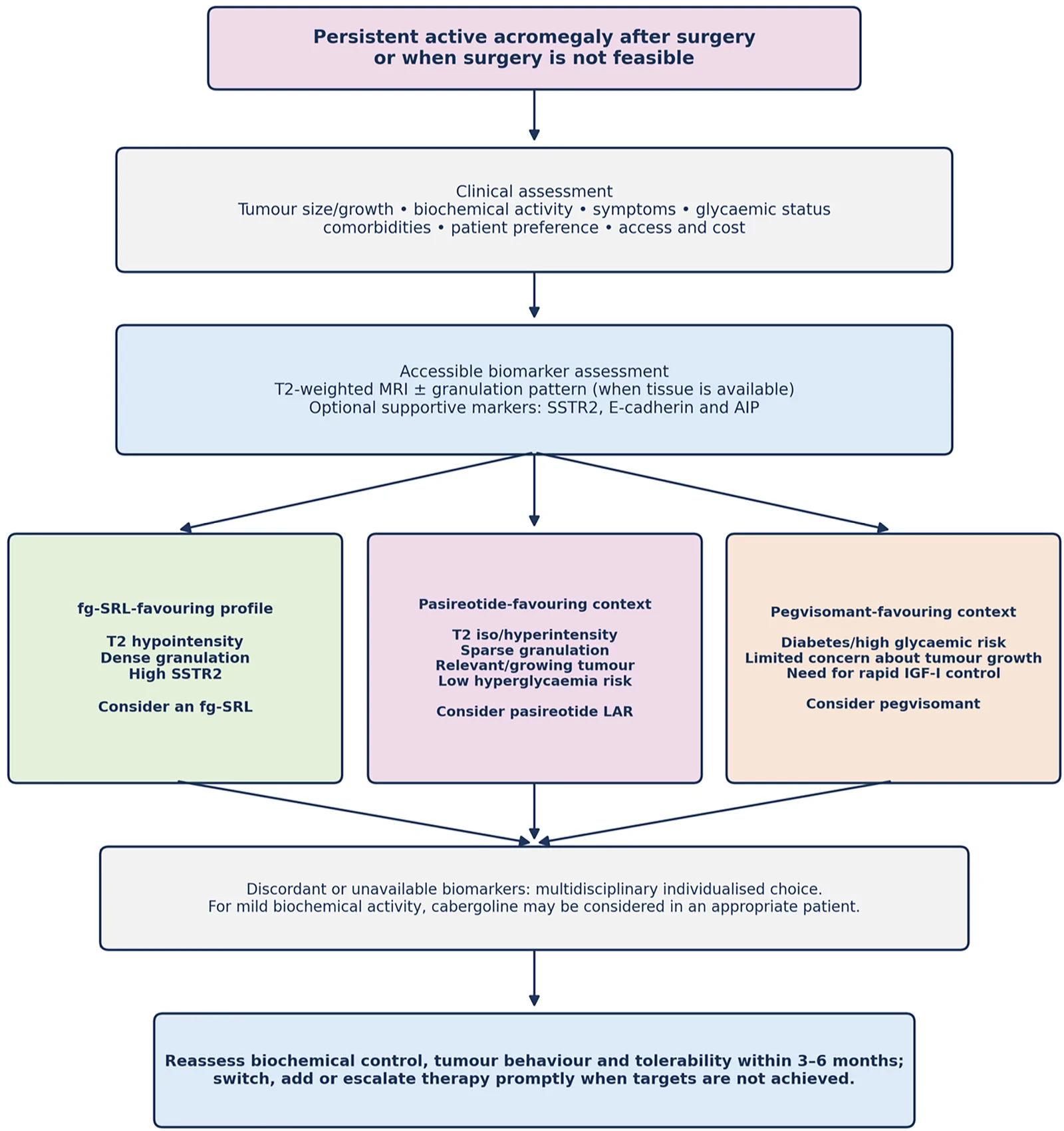
Proposed GCC-adapted algorithm for first-line pasireotide. Based on Puig-Domingo (12), treatment algorithm accommodating use of available tumour response biomarkers in the GCC region.

### Future directions and challenges

Several challenges limit implementation including small sample sizes in biomarker studies, methodological variations between centres, differences in diagnostic technologies and heterogeneity of the data in the literature (11). Solutions include conducting larger multicentre studies, establishing standardised reference centres, and improving access to appropriate equipment and trained personnel (11). The development of machine learning-based prediction models shows promise for improving treatment selection accuracy (10). These models can integrate patient characteristics, tumour features, and molecular markers to provide more precise treatment recommendations than individual biomarkers alone (10).

## Conclusion

The integration of imaging, molecular, and functional biomarkers is transforming acromegaly treatment from “trial and error” towards precision medicine. While current guidelines recommend first-generation somatostatin receptor ligands as first-line therapy, precision medicine approaches open new possibilities for personalised treatment selection, enabling early optimisation of treatments with potential improvements in outcomes and cost-benefits.

Precision medicine implementation in Gulf healthcare systems can leverage widely accessible biomarkers, including T_2_-weighted MRI signal characteristics and histopathological granulation patterns. This combination may provide a strong rationale for first-line pasireotide, potentially eliminating months of inappropriate trial-and-error therapy and reducing time to disease control. In Gulf State healthcare systems, implementing this precision medicine approach could be a valuable opportunity to address regional challenges in acromegaly management, including delayed diagnosis, high metabolic comorbidity burden, and suboptimal control rates.

The future of effective acromegaly management lies in moving beyond trial-and-error approaches to embrace personalised medicine that considers unique molecular, clinical, and patient-specific factors that determine treatment response. Given the currently available tools and regional healthcare infrastructure investment, this vision could be achievable for the Gulf state countries.

## Data Availability

The original contributions presented in the study are included in the article/supplementary material. Further inquiries can be directed to the corresponding author.
